# Transcriptome profiling in rice reveals a positive role for *OsNCED3* in defense against the brown planthopper, *Nilaparvata lugens*

**DOI:** 10.1186/s12864-022-08846-5

**Published:** 2022-09-05

**Authors:** Litong Sun, Jitong Li, Yongyan Liu, Ali Noman, Lin Chen, Jinglan Liu

**Affiliations:** 1 College of Horticulture and Plant Protection, Yangzhou University, Yangzhou, 225009 China; 2 College of Guangling, Yangzhou University, Yangzhou, 225128 China; 3 Department of Botany, Government College University, Faisalabad, 38040 Pakistan; 4 Joint International Research Laboratory of Agriculture and Agri-Product Safety, The Ministry of Education of China, Yangzhou University, Yangzhou, 225009 China

**Keywords:** Abscisic acid, OsNCED3, Nilaparvata lugens, Insect resistance, Transcriptome analysis

## Abstract

9-*cis*-epoxycarotenoid dioxygenase (NCED) is the rate-limiting enzyme for abscisic acid (ABA) biosynthesis in higher plants. In rice, *OsNCED3* was shown to promote ABA synthesis, and improve abiotic stress tolerance, but the function of *OsNCED3* in regulating rice defense against the brown planthopper (*Nilaparvata lugens*; BPH) has been unclear. In this study, several parameters were used to assess rice resistance to BPH, including the average injury level, the functional plant loss index, and electrical penetration graph analysis. Rice lines overexpressing *OsNCED3* (OE) were more resistant to BPH than the wild-type cv. Zhonghua11 (WT). Transcriptome analysis was performed on WT, OE, and a RNAi transgenic line silenced for *OsNCED3*; these three lines were either infested or non-infested with BPH. Seventeen RNA libraries were compared, and most of the differentially expressed genes (DEGs) were upregulated. The number of DEGs in the RNAi line infested with BPH was significantly higher than the OE, and WT lines, and many DEGs were related to the stress response, and biosynthesis of jasmonic acid. This study shows that overexpression of *OsNCED3* in rice improves resistance to BPH, and has potential merit in rice breeding programs.

**Supplementary Information:**

The online version contains supplementary material available at 10.1186/s12864-022-08846-5.

## Introduction

Food security has become a critical issue worldwide due to incremental population growth, and urbanization [1]. The brown planthopper (BPH), *Nilaparvata lugens* Stål, is an insidious pest of rice (*Oryza sativa* L.) in Asia [2], and causes widespread damage, and devastating yield losses in China [3]. BPH is generally controlled by chemical pesticides; however, pesticide abuse can cause serious environmental problems and, may result in insecticide resistance. New pest management strategies that are environmentally-friendly are needed to reduce pesticides usage.

The phytohormone abscisic acid (ABA) controls multiple growth, and developmental processes, including maturation, dormancy, and germination. ABA also regulates the plant response to abiotic stressors, including cold, drought, salinity, and overwatering [4, 5]. In contrast to the phytohormones jasmonic acid (JA), salicylic acid (SA), and ethylene, there is limited research documenting the role of ABA in the plant defense response to insects [6, 7]. It has been reported that ABA deficiency can increase plant susceptibility to herbivory [8–10]. Previous studies showed that exogenous ABA enhanced rice resistance to BPH by promoting callose formation [11, 12]; however, the molecular mechanism underlying ABA-mediated rice defense is poorly understood.

9-*cis*-epoxycarotenoid dioxygenase (NCED) is a rate-limiting enzyme in ABA biosynthesis [13]. *NCED* was first reported in the *Zea mays vp14* mutant, and showed lower endogenous ABA content, higher transpiration rates, and reduced seed germination rates as compared to wild-type plants [14]. *NCED* homologs have been isolated from other plants species, including *Solanum lycopersicum* [15], *Persea americana* [16], *Arabidopsis thaliana* [17, 18], *Malus domestica* [19], and *Brassica napus* [20]. Previous research showed that *AtNCED3* in *Arabidopsis thaliana* was induced during drought stress, and its overexpression improved drought tolerance [21]. In cassava, *MeNCED3* expression was significantly induced by salt stress; this caused an increase in ABA content, and enhanced salt tolerance [22]. There are five homologs in the rice *NCED* gene family [23]; among these, *OsNCED3* expression was elevated in rice undergoing abiotic stress [24]. Salinity, drought, and H_2_O_2_ induced *OsNCED3* expression, thus indicating a critical role for *OsNCED3* in the rice response to abiotic stress [24]. Furthermore, ABA levels increased when *OsNCED3* was expressed in transgenic *Arabidopsis thaliana* [25], and transgenic rice overexpressing *OsNCED3* had higher ABA levels during drought stress [26]. Although ABA improves plant resistance to drought stress, there are relatively few reports on the role of ABA in response to insect stress.

In this study, multiple assessments were performed to evaluate the role of *OsNCED3* in rice resistance to BPH, including the functional plant loss index, average injury level, and electrical penetration graphs. Transcriptome analysis of rice leaf sheaths infested with BPH was performed to further evaluate the role of *OsNCED3* in rice resistance. We show that *OsNCED3* functions in the rice defense response to BPH, which indicates its potential use in developing rice lines with improved resistance to this important pest.

## Materials and methods

### Rice and insect materials

The wide-type rice cv. Zhonghua 11 (ZH11) was used in these experiments. Rice lines overexpressing *OsNCED3* (OE), and or silenced for *OsNCED3* expression by RNA interference (RNAi) were provided by the College of Life Sciences, South China Agricultural University [26]. Seeds were selected, soaked in water, germinated, and then sown in plastic boxes (38 cm long, 22 cm wide, 8 cm high) containing soil. Seedlings were transferred to plastic cups (5 cm diameter, 12 cm high), and supplied with fertilizer, and water. At the age of rice plants in detail (four-leaf stage), rice lines were used in experiments. BPH was collected from China Rice Research Institute (Hangzhou, China), and reared in a greenhouse at Yangzhou University.

### Measurement of *OsNCED3* expression in different rice lines

WT, OE, and RNAi rice lines were used for experiments. Rice leaves (0.5 g) were wrapped in tin foil, placed in liquid nitrogen, and then stored in an ultra-low temperature freezer at -80℃ until needed.

### Determination of rice injury levels after BPH feeding

WT, and OE rice lines (*n* = 15) were selected. A flexible cylinder from a polyvinyl chloride (PVC) sheet was inserted into the soil along the rim of the cup. Third instar BPH nymphs (*n* = 30) were starved for 1 h, and then transferred to the PVC cylinder and, sealed with gauze. The injury level of rice in each plastic cup was checked at seven days, and established rating standards [27] were used to assess injury levels (Table 1).

**Table 1:** Grading of BPH-mediated injury levels in rice seedlings

Injury grade at seedling stage	Standards of International Rice Research Institute
0	Unharmed
1	Victimization is very light
3	First and second leaves of most plants turn yellow
5	Plants become noticeably yellow and dwarfed, or more than half of the plants wither and die
7	More than half of the plants wither and die; remaining plants are severely dwarfed and near death
9	All plants die

### Measurement of functional plant loss indices (FPLI) after BPH feeding

After determining injury levels, rice plants were cut into pieces, then washed, dried at 110℃ for 20 min, and then dried to constant weight at 60℃. Dry weights were measured with a precision electronic balance, and the functional plant loss index (FPLI) was calculated as described previously [28].

$$\mathrm{FPLI}=\;100\;-\frac{\mathrm{Dry}\;\mathrm{weight}\;\mathrm{of}\;\mathrm{injured}\;\mathrm{plant}}{\mathrm{Dry}\;\mathrm{weight}\;\mathrm{of}\;\mathrm{uninjured}\;\mathrm{plants}}\times\;\left(1-\frac{\mathrm{Injured}\;\mathrm{level}}9\right)\times100.$$

### BPH feeding behavior as determined by electrical penetration graphs (EPGs)

Characterization, and quantification of EPG waveforms were performed as described previously [12]. Briefly, individual rice seedlings (four-leaf stage) were transplanted into plastic cups. After a 1-h starvation period, individual 3^rd^ instar BPH nymphs were placed on rice leaf sheaths, and a gold wire (3–5 cm long, 20 μm diameter) was connected from the BPH dorsum to the EPG instrument. The EPG instruments were placed in climate-controlled chambers (25 ± 2℃), and connected to a computer running PROBE software (EPG-System, Wageningen, the Netherlands). BPH with a gold wire attached to the dorsum by conductive silver glue was then allowed to probe the rice sheath through the parafilm. The gold wire from each insect, and a copper wire (0.1 mm dia.) immersed in the food were linked to a model CR-8 DC-EPG amplifier (Wuhan Pusaisi Electronic Technology Co., Ltd.). Data were analyzed using ANA v. 3.0 software (Wageningen University). EPG recordings were carried out for 6 h per insect per plant, with 10 replicates for each treatment.

### RNA library construction and transcriptome sequencing

Six seedlings for each of the WT, OE, and RNAi rice lines were selected at the tillering stage. Plants used for BPH feeding were covered with a handmade plastic cover, and sampling was carried out at 12 h after BPH feeding based on our previous work [12]. plants without BPH feeding were used as controls. Each treatment was replicated three times. Samples (0.2 g) were wrapped in foil, frozen in liquid nitrogen, and then stored in an ultralow freezer at -80℃.

RNA library construction, and quality control were conducted as described previously [29]. Total RNA was extracted from samples using the Total RNA Kit (Tiangen, Beijing, China). Samples were concentrated using oligo (dT) magnetic adsorption, and used as templates for first-strand cDNA synthesis; second-strand cDNA was synthesized, and purified with AMPure XP beads. cDNA libraries were analyzed with Qubit 2.0, and Agilent 2100 prior to sequencing with the Illumina HiSeq 2500 High-throughput Sequencing System (located at Genepioneer Biotechnologies Co, Ltd., Nanjing, China). The clean data reads that were obtained after quality control were compared with the reference genome (ftp://ftp.ensemblgenomes.org/pub/plants/release-44/fasta/ oryza_sativa/dna/) to obtain mapped data reads for subsequent transcript assembly, and expression calculation. Multiple comparisons with the reference genome were made using Cufflinks (http://cole-trapnell-lab.github.io/cufflinks/). The number of fragments per kilobase of exon per million fragments (FPKM) was calculated for gene expression analysis. DEGs were defined as those fulfilling the following requirements: *p*-adjust ≤ 0.05, and default difference multiple = 2. DAVID24 was used to analyze for over-representation of gene classes. Differential expression analysis was conducted using the expression level of genes in sample groups, and GO functional annotation, and KEGG pathway enrichment analysis were performed on differentially expressed genes (DEGs).

### Identification of DEGs and verification by qRT-PCR

DEGs were detected using DESeq2 software, and log_2_ fold change ≥ 1, and false discovery rates (FDR) < 0.05 were used as screening criteria. Fold change represents the ratio of expression among groups, and FDRs were obtained by correcting the significant *P* values. Six DEGs were selected for real-time quantitative PCR, and the accuracy of transcriptome sequencing was verified. HiScript ®Q RT SuperMix for qPCR (+ gDNA wiper) (Vazyme) was used for reverse transcription, and Primer Premier 5.0 was used for primer design. Quantitative PCR was performed using the ChamQTM SYBR ®Color qPCR Master Mix Kit (Vazyme). The mixed solution was added to 96-well plates, and the reaction mixture contained primers of the target gene (0.8 μl), 2 × ChamQ SYBR Color qPCR Master Mix (10 μl), and template (2 μl). cDNA from rice sheaths was used as template, *ActinI* was selected as the internal reference, and each sample was tested thrice. The relative expression of each gene was calculated by the 2^−ΔΔCt^ method [30].

### Statistical analysis

The statistical significance of differences between treatments was determined by analysis of variance (ANOVA; Systat Inc.) followed by Duncan´s multiple range test for multiple comparisons. For ANOVA, data were analyzed directly if normally distributed; data that were not normally distributed were transformed to ensure homogeneity of variances among different groups. Data were denoted as means ± SE, and analyzed using SPSS 11.0 software (SPSS).

## Results

### Measurement of *OsNCED3* expression levels in different rice lines

**Fig. 1:**
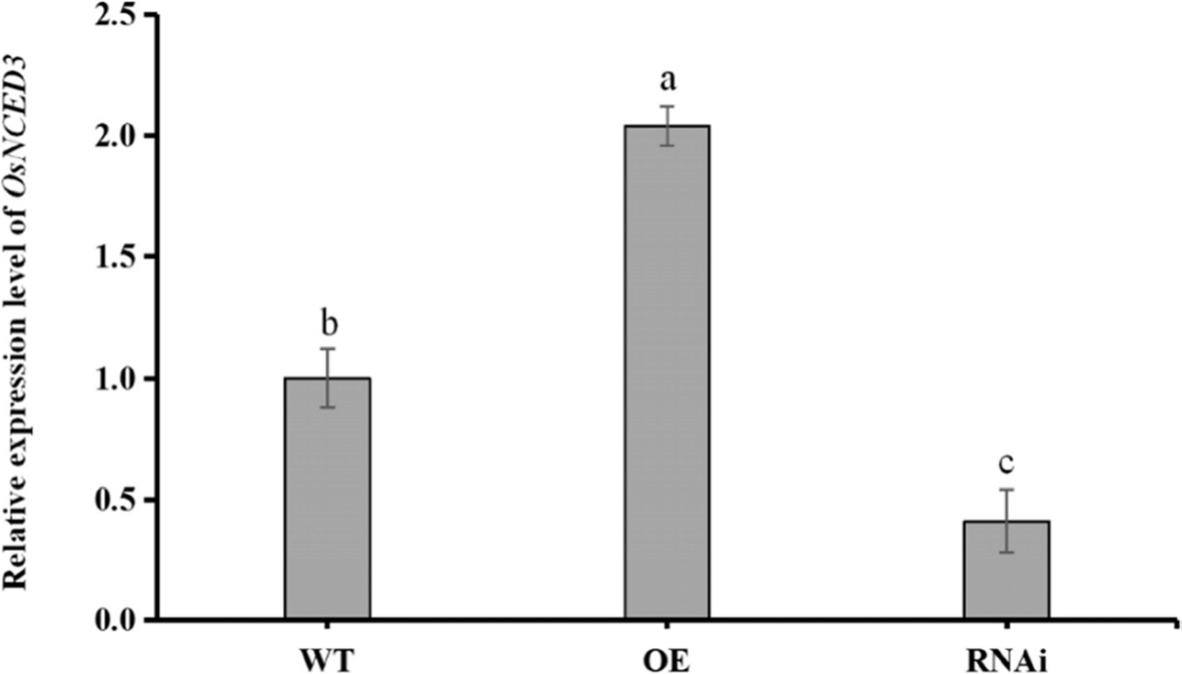
Measurement of OsNCED3 expression in WT, OE, and RNAi rice lines. Relative expression levels were determined by qPCR. Error bars represent means ± SE, and three independent experiments were conducted with three or more replicates. Bars with different letters indicate significant differences at *P* < 0.05 (Duncan´s multiple range test)

The expression of *OsNCED3* in WT, OE, and RNAi rice lines was measured by RT-qPCR. Expression of *OsNCED3* in OE rice increased by 106.30% as compared to expression levels in the WT, whereas *OsNCED3* expression in RNAi rice was downregulated by 66.60% (Fig. 1). These results indicated that overexpression, and knock-down experiments were successful.

### Determination of injury levels, FPLI, and BPH feeding behavior

**Fig. 2:**
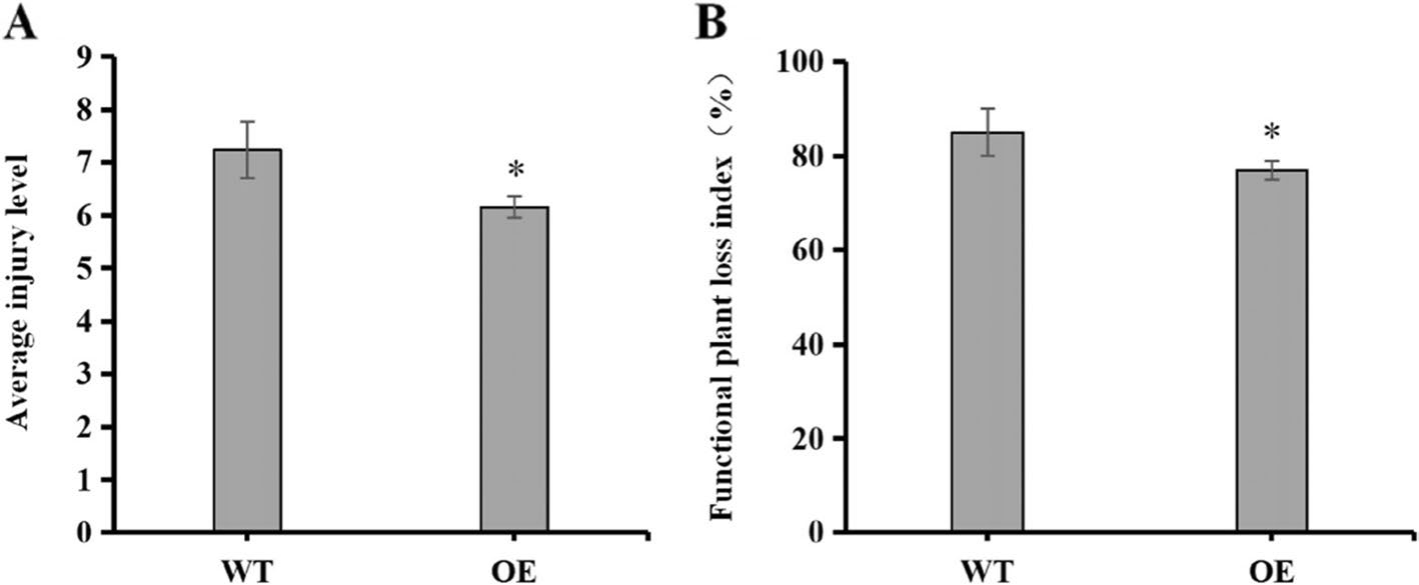
Average injury levels, and FPLI after BPH feeding on wild-type (WT) rice, and transgenic rice overexpressing (OE) OsNCED3. BPH (*n* = 30) were allowed to feed on WT, and OE rice for 7 d, and injury, and FPLI values were then obtained. Panels: **A** Average injury levels, and **B** FPLI. Error bars represent means ± SE, where *n* = 3, and experiments contained three or more replicates. Columns with asterisks (*) indicate significant differences at *P* < 0.05 (Student T-test)

In OE rice, the mean damage levels, and FPLI values were 14.64%, and 9.41% lower than the WT, respectively (Fig. 2).

**Fig. 3:**
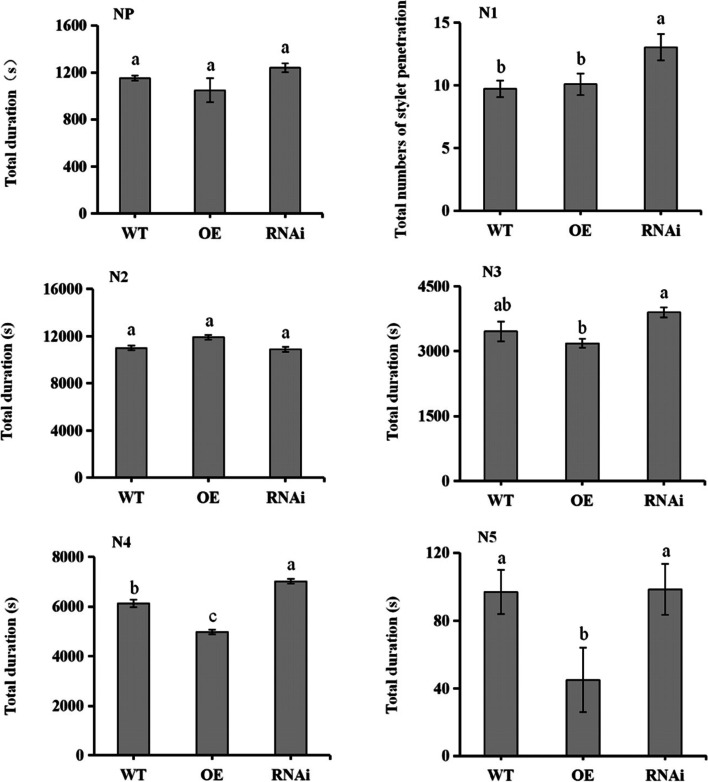
Analysis of BPH feeding behavior using electrical penetration graph (EPG) analysis. Abbreviations: WT, wild-type rice; OE, rice overexpressing OsNCED3; and RNAi, OsNCED3-silenced rice. A single BPH was allowed to feed on an individual rice plant, and the effective feeding time was 6 h. Panels: NP, the duration of the non-probing stage; N1, duration of probing; N2, duration of stylet movement in vascular bundles; N3, duration of stylet movement outside phloem cells; N4, duration of sap ingestion in the phloem; and N5, duration of water ingestion in xylem. Error bars represent the means ± SE where *n* = 3; experiments contained three or more replicates. Columns labeled with different letters are significantly different at *P* < 0.05 (Duncan´s multiple range test)

BPH feeding behaviors in WT, OE, and RNAi rice lines using EPG are shown in Fig. 3. There were no significant differences in the duration of the nonprobing (NP) waveform among the three rice lines. With respect to the duration of probing (N1 waveform), the frequency of the waveform in RNAi rice was significantly higher than the WT, and OE lines. There was no significant difference in the duration of the N2 waveform (duration of stylet movement in vascular bundles) among the three rice plants lines. However, the N3 waveform, which indicates the duration of stylet movement outside phloem cells, was significantly lower in the OE vs. the RNAi line. The N4 waveform indicates the duration of sap ingestion in the phloem, and this was significantly different in the three rice lines. When compared with the WT, the duration of the N4 wave in OE rice was significantly reduced by 20.13%, whereas it increased in the RNAi line by 16.49%. The N5 waveform, which indicates the ingestion of xylem fluids by BPH, was not significantly different in the WT, and RNAi rice lines. The N5 waveform was 53.61% lower in OE rice as compared to WT, and was 118.89% higher in the RNAi line than OE rice (Fig. 3). These results indicate that overexpressed *OsNCED3* can improve BPH resistance in rice.

### Transcriptome data

The quality of transcriptome sequencing data is shown. After quality control, a total of 128.00 Gb of clean data was obtained, and the clean data in each sample was 6.19 Gb or above. Clean reads of individual samples were compared with the reference genome, and the comparison efficiency was 91.36% or greater. The Q30 percentages in the 17 rice samples exceeded 89.29%, indicating that the data are highly usable. The GC content in clean reads was between 48.81%—52.25%. Overall, these statistics indicate that the sequencing results were qualified, and could be used for further analysis.

### DEGs in OE and RNAi rice lines after BPH feeding

**Fig. 4:**
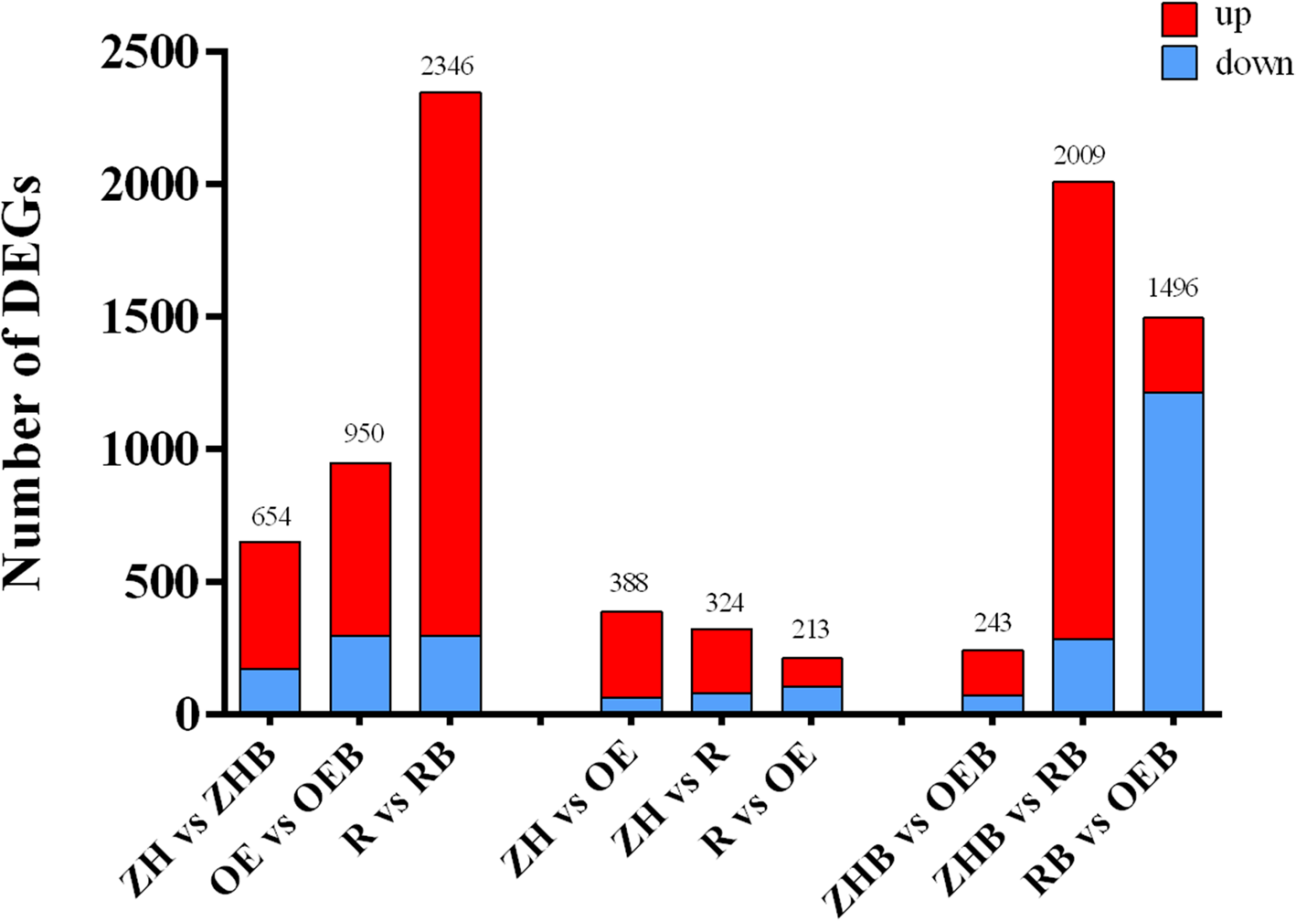
Up- and down-regulated DEGs in different rice lines with or without BPH feeding. Red and blue shading represent up- and down-regulated DEGs, respectively. Column heights represent the number of up- or down-regulated DEGs. Abbreviations: ZH: WT without BPH feeding; ZHB, WT with BPH feeding; OE, OE rice without BPH feeding; OEB, OE rice with BPH feeding; R, RNAi rice without BPH feeding; RB, RNAi rice with BPH feeding

DEGs in the three rice lines were identified in the presence, and absence of BPH feeding (Fig. 4). Statistical analysis of DEGs in the three rice lines was carried out, and the number of up- and down-regulated genes in each group was obtained (Fig. 4). The first three columns show a comparison of rice lines with BPH feeding (ZHB, OEB, RB), and without BPH feeding (ZH, OE, R). When rice lines were compared in the presence, and absence of BPH feeding, the total number of DEGs in ZH vs. ZHB was 654,950 in OE vs. OEB, and 2346 in R vs. RB. The number of DEGs was smallest when comparing RNAi, and OE groups without BPH feeding, which was 388 (R vs. OE); the number of DEGs was highest in the RNAi lines with, and without BPH feeding, which was 2346 (R vs. RB). The data also show that the number of DEGs in the RNAi / WT comparison with BPH feeding (ZHB vs. RB) was much higher than the comparison without BPH feeding (ZH vs. R) (Fig. 4).

Analysis of DEGs showing 2–sevenfold-change differences revealed a large number of up- and down-regulated DEGs in the four comparative groups (Table 2), and more genes were up- vs. down-regulated. Fold-change differences were highest in RNAi rice lines infested with BPH; for example, 1724 DEGs showed fold-change values ≥ 2, which was significantly more than other compared groups (Table 2). These results show that only a few DEGs were commonly expressed in the different rice lines infested with BPH.

**Table 2:** Statistical results of differentially expressed genes subjected to various difference multiples (*p* < 0.05)

Fold- change	Downregulated				Upregulated			
	ZH vs. OE	ZHB vs. OEB	ZH vs. R	ZHB vs. RB	ZH vs. OE	ZHB vs. OEB	ZH vs. R	ZHB vs. RB
≥ 2	65	72	81	285	323	171	243	1724
≥ 3	28	35	39	81	164	127	161	786
≥ 4	18	19	27	45	126	109	124	464
≥ 5	16	16	17	29	107	99	108	331
≥ 6	15	14	11	24	93	85	93	263
≥ 7	15	11	10	23	85	82	88	213

### Venn diagrams of DEGs

**Fig. 5:**
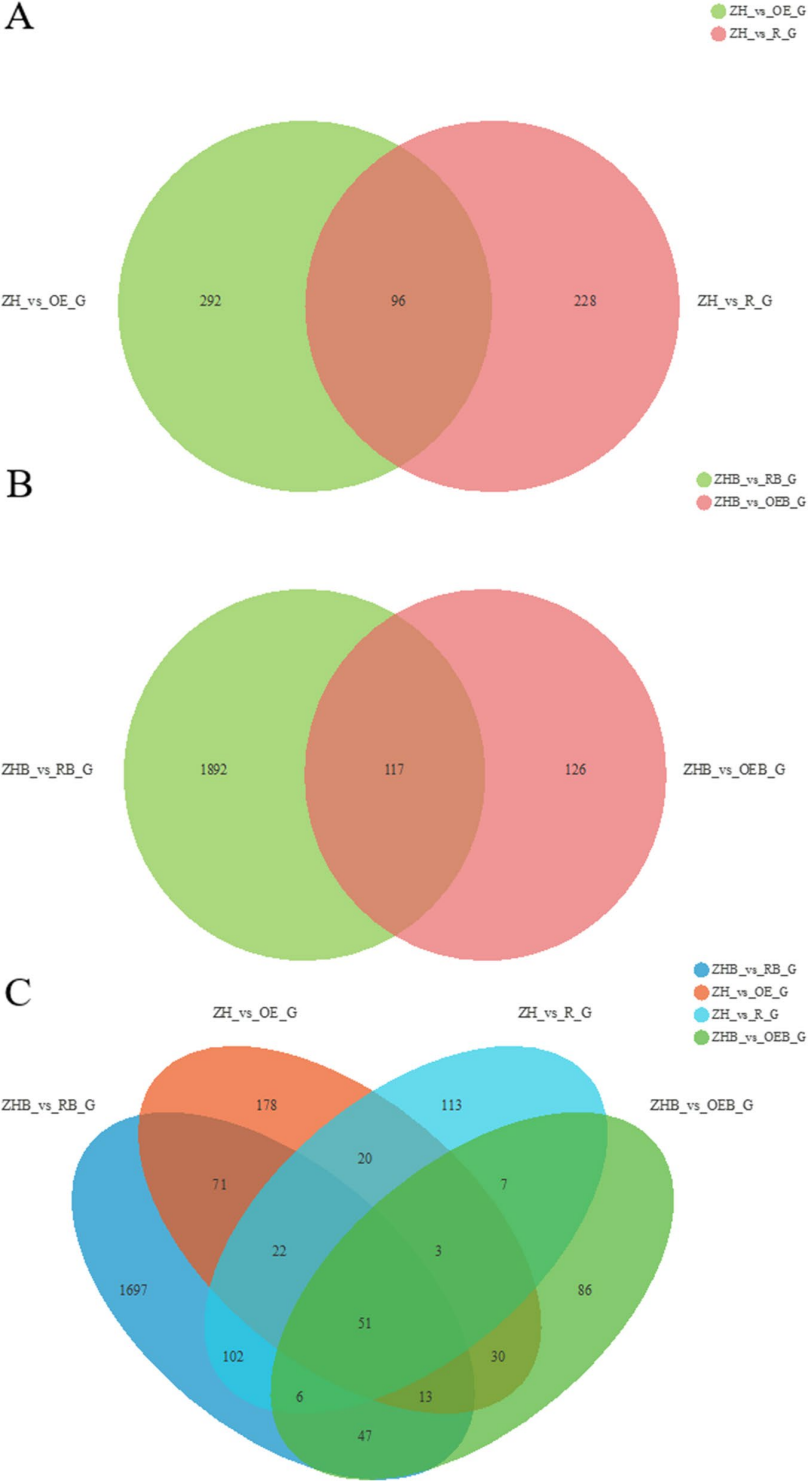
Venn diagrams of DEGs in different comparative groups. **A** ZH vs. R_G represents DEGs in the ZH11 (WT), and RNAi rice group. ZH vs. OE_G represents DEGs in the ZH11, and OE rice group. **B** ZHB vs. OEB_G shows DEGs in the ZH11, and OE rice group after BPH feeding; and ZHB vs. RB_G shows DEGs in the ZH11, and RNAi rice group after BPH feeding. **C** DEGs in the four compared groups

In the absence of BPH feeding, the number of overlapping DEGs in the WT (ZH) vs. OE, and WT vs. RNAi groups were 96, and these accounted for 29.63%, and 24.74% of the total number of DEGs, respectively (Fig. 5A). After BPH feeding, there were 117 overlapping DEGs in ZHB vs. OEB, and ZHB vs. RB groups, and these represented 48.15%, and 5.82% of the WT DEGs, respectively (Fig. 5B). When the four compared groups were analyzed, 51 overlapping DEGs were identified, accounting for 2.54%, 20.99%, 15.75%, and 13.14% of the total number of DEGs in the control groups (Fig. 5C). These results show that only a few DEGs were common to the different rice lines infested with BPH.

### GO functional analysis of DEGs

**Fig. 6:**
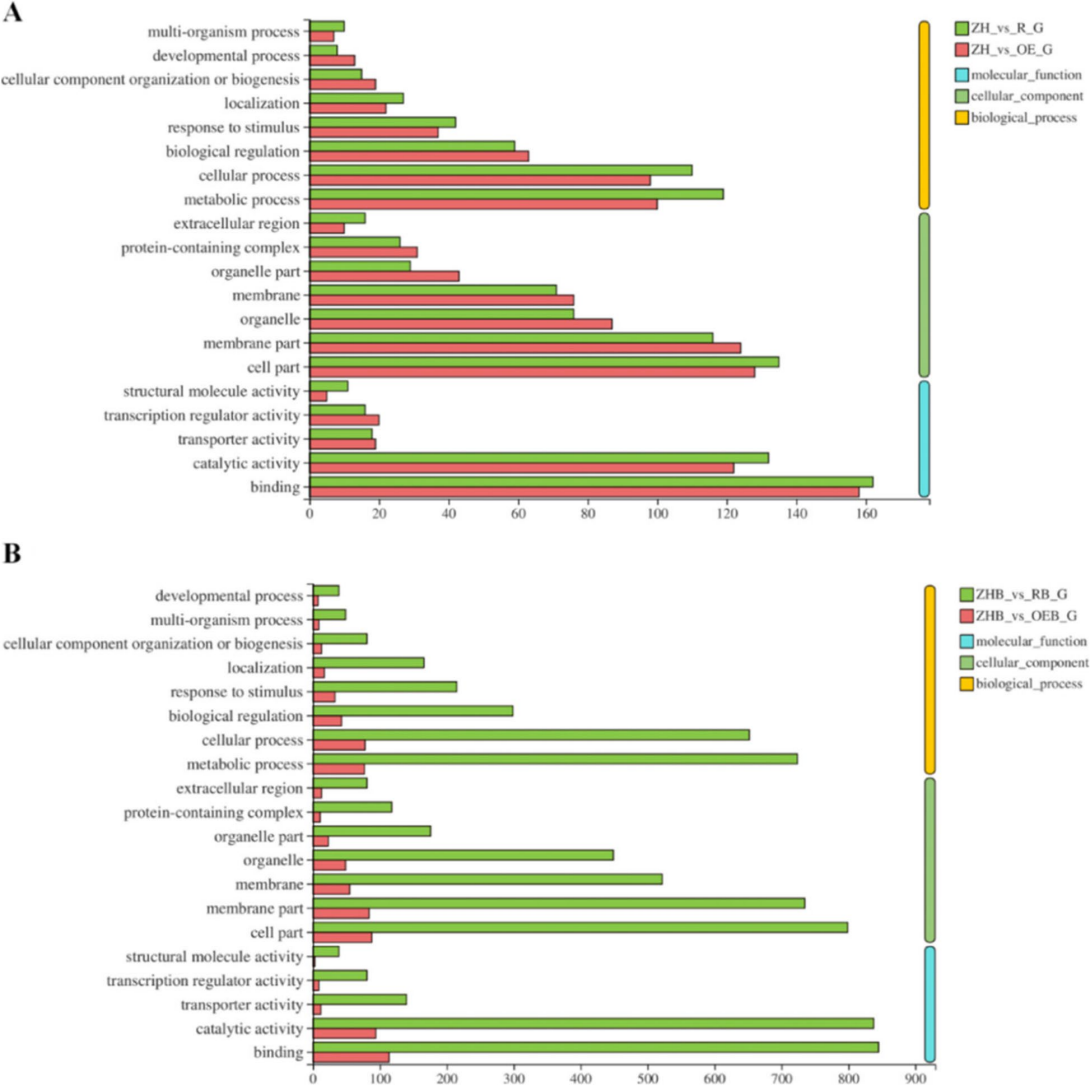
GO annotation of DEGs in different groups. **A** ZH vs R_G represents DEGs in the ZH11 (WT), and RNAi rice group; and ZH vs OE_G shows DEGs in the ZH11, and OE rice groups. **B** ZHB vs OEB_G shows DEGs in the ZH11 and OE rice group after BPH feeding; and ZHB vs RB_G indicates DEGs in the ZH11, and RNAi rice group after BPH feeding

In the absence of BPH feeding, DEGs in the ZH (WT) vs. OE, and the ZH vs. R (RNAi) groups were highly represented in the GO molecular function "binding" (GO:0,005,488) category (Fig. 6A). The number of DEGs annotated as "catalytic activity" (GO:0,003,824), "cell part" (GO:0,044,464), "membrane part" (GO:0,016,020), "metabolic process" (GO:0,008,152), and "cellular process" (GO:0,009,987) all exceeded 100 (Fig. 6A).

After BPH feeding, the number of DEGs annotated to GO functions increased significantly in RNAi rice, and the highest proportion (*n* = 845) were assigned to "binding", followed by "catalytic activity", "cell part", and "metabolic process". The number of DEGs in the top six GO pathways included "biological regulation", "metabolic process", "membrane part", "cell part", "binding", and "catalytic activity", and exceeded 600 (Fig. 6B). After BPH feeding, the number of DEGs annotated to "binding" in OE rice lines was 114, followed by "metabolic process", "cell process", and "cell part". However, there were less than 100 DEGs in each GO pathway in WT vs. OEB_G, and the number of DEGs in each GO pathway was significantly less than the number in RNAi rice lines (Fig. 6B).

### KEGG functional analysis

**Fig. 7:**
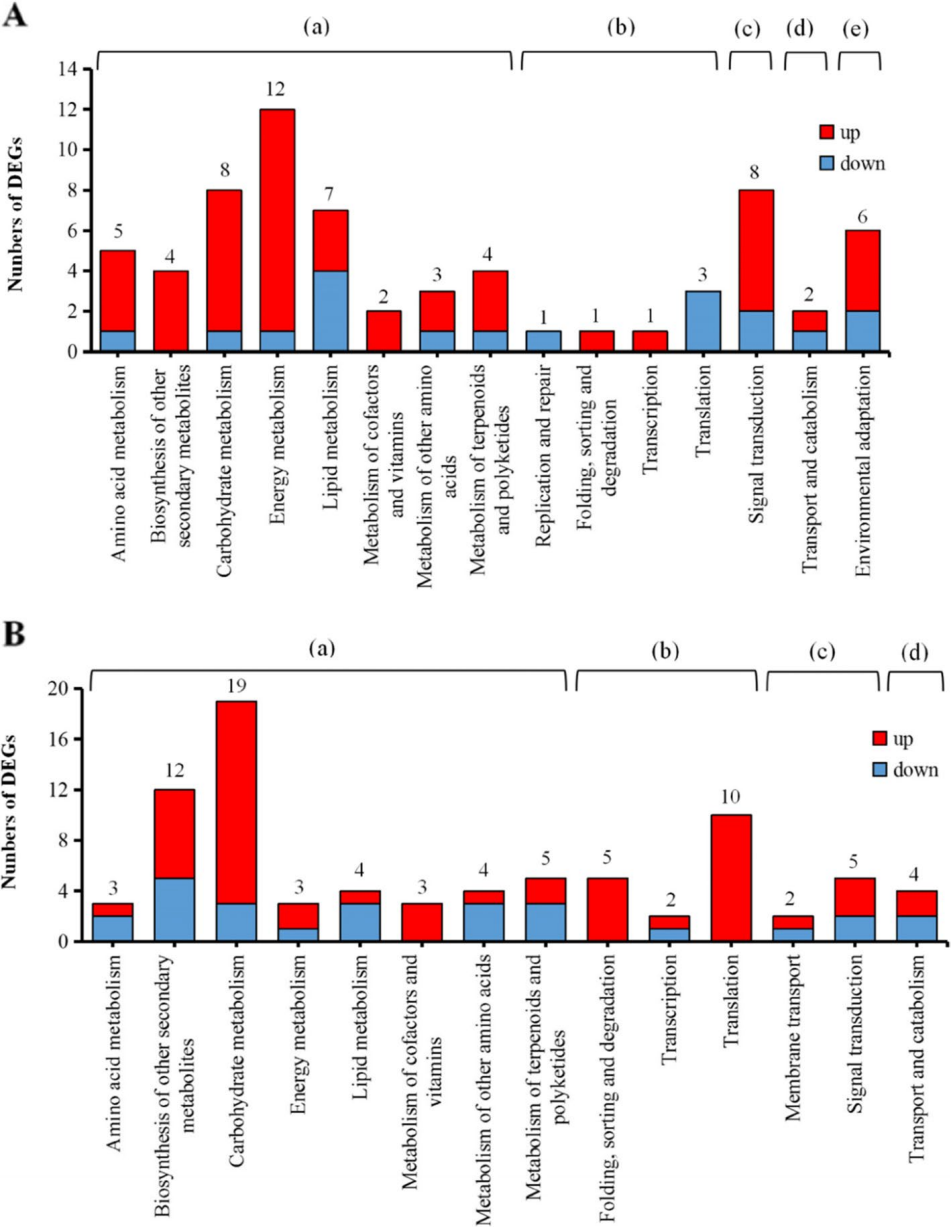
KEGG annotation of DEGs in different comparative groups in the absence of BPH feeding. **A** KEGG pathways represented in up- and down-regulated DEGs of OE rice without BPH feeding. **B** KEGG pathways represented in up- and down-regulated DEGs in RNAi rice without BPH feeding. Pathway categories include: (a) metabolism; (b) genetic information processing; (c) environmental information processing; (d) cellular processes; and (e) organismal systems

In the absence of BPH feeding, DEGs in the WT vs. OE lines were concentrated in 15 KEGG pathways, including amino acid metabolism, biosynthesis of other secondary metabolites, carbohydrate metabolism, energy metabolism, lipid metabolism, metabolism of cofactors, and vitamins, metabolism of other amino acids, metabolism of terpenoids, and polyketides, signal transduction, transport, and catabolism, and environmental adaptation pathways (Fig. 7A). The largest number of DEGs (*n* = 12) were involved in the energy metabolism pathway, followed by the carbohydrate metabolism pathway (*n* = 8), and signal transduction (*n* = 8).

The DEGs in ZH (WT) vs. RNAi rice were concentrated in 14 KEGG pathways in the absence of BPH feeding. These included amino acid metabolism, biosynthesis of other secondary metabolites, carbohydrate metabolism, energy metabolism, lipid metabolism, metabolism of cofactors, and vitamins, metabolism of other amino acids, metabolism of terpenoids, and polyketides, signal transduction, transport, and catabolism, and environmental adaptation pathways (Fig. 7B). The most highly-represented pathway was carbohydrate metabolism (*n* = 19 DEGs), followed by biosynthesis of other secondary metabolites (*n* = 12).

**Fig. 8:**
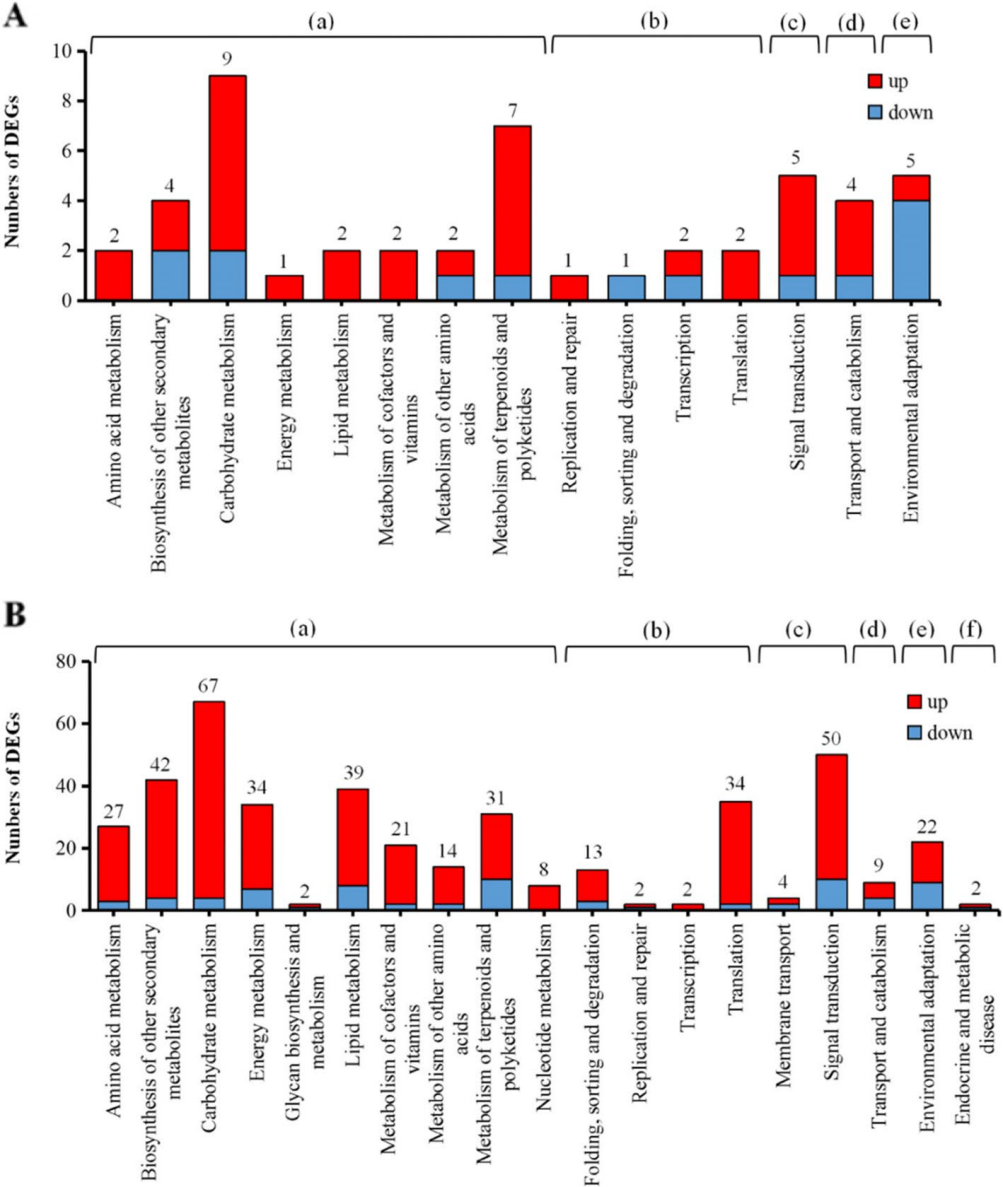
KEGG annotation of DEGs in different comparative groups with BPH feeding. **A** KEGG pathways represented in up- and down-regulated DEGs of OE rice with BPH feeding. **B** KEGG pathways represented in up- and down-regulated DEGs in RNAi rice with BPH feeding. Pathway categories include: (a) metabolism; (b) genetic information processing; (c) environmental information processing; (d) cellular processes; (e) organismal systems; and (f) endocrine, and metabolic disease

After BPH feeding, the assignment of DEGs to KEGG pathways in the OE, and RNAi lines were similar to those without BPH feeding. The highest number of DEGs were in the carbohydrate metabolism pathway (*n* = 9), followed by metabolism of terpenoids, and polyketides (*n* = 7) (Fig. 8A). After BPH feeding on RNAi rice, DEGs were distributed in 19 KEGG pathways. This includes four new pathways that were not represented in the absence of BPH feeding, including glycan biosynthesis, and metabolism, nucleotide metabolism, environmental adaptation, and endocrine, and metabolic disease. The carbohydrate metabolism pathway was the most highly represented with 67 DEGs, followed by the signal transduction pathway (*n* = 50) (Fig. 8B).

### qRT-PCR verification of transcriptome results

**Fig. 9:**
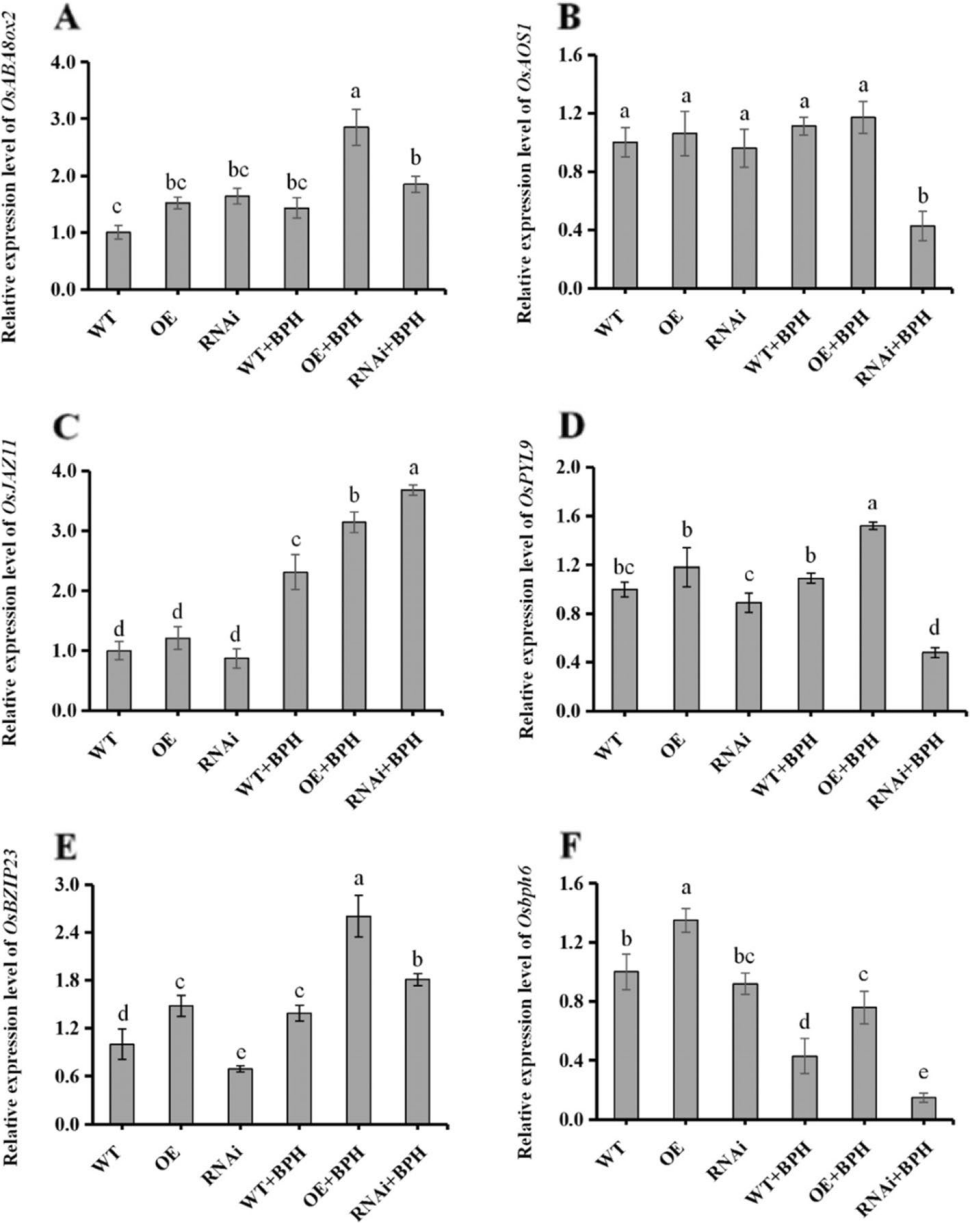
Verification of transcriptome results by qRT-PCR. Six genes related to ABA, JA, and rice defense were selected, and relative expression levels were determined by qRT-PCR, and compared with transcriptome results. Genes included the following: (**A**) OsABA8ox2 encoding ABA 8’-hydroxylase (LOC_Os08g36860); (**B**) OsAOS1 encoding allene oxide synthase (LOC_Os03g55800); (**C**) OsJAZ11 encoding jasmonate ZIM-domain protein (LOC_Os04g32480); (**D**) OsPYL9 encoding pyrabactin resistance-like abscisic acid receptor (LOC_Os06g36670); (**E**) OsBZIP23 encoding bZIP transcription factor (LOC_Os02g52780) l; and (**F**) Osbph6 encoding leucine rich repeat family protein (LOC_Os04g35210). Error bars represent means ± SE (*n* = 3); three or more independent experiments were conducted. Columns with different letters indicate significance at *P* < 0.05 using Duncan´s multiple range test

Six DEGs related to hormone pathways, and insect defense were selected for qRT-PCR analysis. *OsABA8ox2* (Os08g36860), and *OsPYL9* (Os06g36670) encode genes involved in ABA degradation, and an ABA receptor. The results show that the expression of the two genes in OE rice lines increased significantly after BPH feeding (Fig. 9a, d). *OsAOS1* (Os03g55800) is involved in JA biosynthesis, and *OsJAZ1* (Os04g32480) is a transcriptional inhibitor of JA. The expression of *OsJAZ1* increased while that of *OsAOS1* decreased in RNAi rice lines infested with BPH (Fig. 9b, c). The relative expression of the rice stress tolerance gene *OsbZIP23* (Os02g52780), and the BPH resistance gene *Osbph6* (Os04g35210) in OE rice was consistently higher than the WT (Fig. 9e, f). The results of qRT-PCR were consistent with transcriptome analysis, and the correlation between the RNA-Seq, and qPCR results support the reliability of our results.

## Discussion

EPG analysis is used to explore insect feeding behavior, and can accurately record the stylet position in host tissue [31]; therefore, EPG technology can be used as a bioassay for evaluating insect resistance in plants [32, 33]. In the current study, results obtained from EPG analysis, average injury levels, and FPLI values showed that *OsNCED3* overexpression increased resistance to BPH in rice. The N4 waveform indicates the duration of sap ingestion in the phloem sap, and is an important marker for measuring plant resistance. In this study, the duration of the N4 wave decreased in OE rice after BPH feeding but increased in RNAi rice lines (Fig. 3). These results imply that *OsNCED3* overexpression was not conductive to BPH feeding in the phloem, and confirm that *OsNCED3* is an important indicator of BPH resistance in rice.

In preliminary investigations, several different time points were examined after BPH feeding (e.g. 2, 4, 8, 12, 24, and 48 h). We choose the 12 h time point because many enzymes and hormones involved in plant defense are altered at this sampling time, including superoxide dismutase, peroxidase, catalase, polyphenol oxidases, and JA, SA, and ABA.

The number of DEGs in WT, OE, and RNAi rice lines were impacted by BPH feeding. The number of DEGs in RNAi rice was significantly higher than the OE rice line, especially after BPH feeding (Fig. 4). In this context, our findings are consistent with a previous report showing that a susceptible rice cultivar had a larger number of DEGs in response to BPH feeding than a resistant rice cultivar [34]. Our results suggest that loss of *OsNCED3* function affects gene regulation in rice after BPH feeding, leading to an upregulation of many genes.

The DEGs identified in OE rice were related to the synthesis of lignin, chitin, and serotonin. *OsCCR17* encodes cinnamoyl-CoA reductase 17, and is involved in the biosynthesis of lignin, and other secondary metabolites. Prior studies have shown that rice homologs encoding cinnamoyl-CoA reductase were induced in response to biotic, and abiotic stress, including pathogen ingress, UV irradiation, and high salinity, thus indicating a role in defense-related processes [35]. *CmMYB15*, which encodes a transcription factor in chrysanthemums, binds to the upstream region of lignin biosynthesis genes, and enhances resistance to aphids [36].

In the carbohydrate metabolism pathway, we identified three chitinase-encoding genes, namely *Oscht1*, *Oscht4,* and *Oscht5*; *Oscht1* expression was elevated in OE rice as compared with the WT in both the presence, and absence of BPH feeding. *Oscht4* and *Oscht5* increased significantly in OE rice after BPH feeding. Chitinase has a role in plant defense, and the overexpression of chitinase genes in pepper, and other transgenic plants enhanced disease resistance, and stress tolerance [37, 38]. Rajendran et al. (2011) demonstrated that increased chitinase activity was correlated with reduced numbers of aphids, which suggests a role for chitinase in defense against both insects, and pathogens [39]. Chitinase occurs in various plant tissues, including leaves, fruits, seeds, and roots; it is regarded as a defense-related enzyme that confers some protection against fungi, and pests [40].

*OsTDC1* encodes tryptophan decarboxylase, which catalyzes the conversion of tryptophan to tryptamine. Tryptamine is further metabolized by tryptamine 5-hydroxylase to form 5-hydroxytryptamine/serotonin [41]. In our study, *OsTDC1* was present in both amino acid, and secondary metabolite pathways after BPH feeding, which suggests a role in the rice defense response. In a related study, increasing levels of 5-hydroxytryptamine in rice mutant lines resulted in decreased resistance to pests [42, 43]

JA is considered a key phytohormone in plant resistance to insects. In our study, the JA synthesis genes *OsHI-LOX* (13-lipoxygenase), *OsLOX7* (lipoxygenase 7), and *OsOPR10* (12-oxo-phytodienoic acid reductase 10) were highly expressed in OE plants infested with BPH. Loss of function studies with *OsHI-LOX* made rice plants more vulnerable to chewing insects but enhanced resistance to phloem-feeding insects [44]. *OsOPR10* was upregulated in OE rice, suggesting a role in rice defense. OPRs are involved in JA biosynthesis, and respond to various biologic, and abiotic stressors, including mechanical damage, salinity, plant signal molecules, and pathogen infection [45].

RT-qPCR results showed that expression of the ABA degradation gene, *OsABA8ox2*, was significantly increased in OE rice. The expression of *OsPYL9*, which encodes an ABA receptor, was significantly higher in OE rice than RNAi rice. *OsPYLs* positively regulate ABA responses during seed germination, and overexpression of *OsPYL9* significantly improved drought, and cold tolerance in rice [46]. We speculate that ABA content in OE rice increased after BPH feeding, and excessive ABA was hydrolyzed.

Expression of the JA biosynthesis gene *OsAOS1* was inhibited in RNAi rice infested with BPH, which likely contributes to a decrease in JA synthesis. *OsJAZ11* was upregulated in WT, OE, and RNAi lines infested with BPH, and may suppress signal transduction in the JA pathway. JAZ proteins negatively regulate plant defense against biotic, and abiotic stress [47, 48]. Our qPCR data suggest that *OsNCED3,* and *OsBZIP23* act synergistically with respect to BPH defense. *OsbBZIP23* was previously shown to function in abiotic stress tolerance in rice [49]. *OsBph6* is a broad-spectrum resistance gene that confers resistance to various BPH biotypes, and the white-backed planthopper via antibiosis, and protaxis. *OsBph6* did not negatively impact rice growth or yield, and conferred a high level of resistance to both indica, and japonica rice [50]. The expression of *Osbph6* in OE rice was significantly higher than the WT in both the presence, and absence of BPH (Fig. 9F), which suggested that OE rice had higher resistance to BPH.

## Conclusions

Based on our results, we conclude that *OsNCED3* improves rice resistance to BPH. *OsNCED3*-mediated defense might arise through increased synthesis of lignin, chitin, and other defense related substances, such as ABA, and JA. The potential use of *OsNCED3* in rice breeding programs has merit but needs further validation.

## Supplementary Information

**Additional file 1:** **Table S1.** RNA-seq quality of seventeen samples. **Table S2.** Primer sequences used in real-time quantitative PCR. **Table S3.** Enriched genes involved in different pathway.

## Data Availability

All data generated or analyzed during this study are included in this published article. Jinglan Liu should be contacted to request the data from this study. The data that support the findings of this study have been deposited into CNGB Sequence Archive (CNSA) [51] of China National Gene Bank Data Base (CNGB db) [52] as accession number CRA007043.

## Abbreviations

ABA: Abscisic acid
JA: Jasmonic acid
ET: Ethylene
BPH: Brown planthopper
NCED: 9-Cis-epoxycarotenoid dioxygenase
OE: Overexpression
RNAi: RNA interference
WT: Wild-type
EPG: Electrical penetration graph
FPLI: Functional plant loss index
DEGs: Differentially expressed genes
ZH11: Zhonghua 11
PVC: Polyvinyl chloride
FPKM: Fragments per kilobase of transcript per million mapped reads
RT-qPCR: Real-time quantitative polymerase chain reaction
GO: Gene Ontology
KEGG: Kyoto Encyclopedia of Genes, and Genomes
