# Supplementary material for: Transcriptome profiling in rice reveals a positive role for OsNCED3 in defense against the brown planthopper, Nilaparvata lugens
Source: BMC Genomics. 2022 Sep 5;23:634. doi: 10.1186/s12864-022-08846-5 (PMC9446700; doi:10.1186/s12864-022-08846-5)
Supplement: Supplementary file 1 — Additional file 1: Table S1. RNA-seq quality of seventeen samples. Table S2. Primer sequences used in real-time quantitative PCR. Table S3. Enriched genes involved in different pathway. [file 12864_2022_8846_MOESM1_ESM.docx]

**Table S1** RNA-seq quality of seventeen samples.

| Sample | Raw reads | Raw bases | Error rate (%) | Q20(%) | Q30(%) | GC content (%) |
| --- | --- | --- | --- | --- | --- | --- |
| OEB1 | 48042250 | 7206337500 | 0.0264 | 97.32 | 92.96 | 48.81 |
| OEB2 | 54509412 | 8176411800 | 0.0273 | 97 | 92.16 | 50 |
| OEB3 | 54101264 | 8115189600 | 0.0265 | 97.32 | 92.92 | 51.15 |
| OE1 | 52051592 | 7807738800 | 0.0311 | 95.45 | 89.29 | 52.17 |
| OE2 | 41267608 | 6190141200 | 0.0262 | 97.42 | 93.12 | 52.25 |
| OE3 | 49202446 | 7380366900 | 0.0263 | 97.39 | 93.06 | 51.65 |
| R1 | 47863938 | 7179590700 | 0.0268 | 97.2 | 92.59 | 49.89 |
| R2 | 51217950 | 7682692500 | 0.0265 | 97.31 | 92.84 | 51.76 |
| R3 | 47164764 | 7074714600 | 0.0266 | 97.26 | 92.75 | 52.02 |
| RB1 | 59765034 | 8964755100 | 0.026 | 97.5 | 93.37 | 52.15 |
| RB2 | 48530162 | 7279524300 | 0.0259 | 97.55 | 93.42 | 51.65 |
| RB3 | 48435258 | 7265288700 | 0.027 | 97.1 | 92.46 | 52.17 |
| ZH1 | 52793434 | 7919015100 | 0.026 | 97.54 | 93.33 | 50.95 |
| ZH2 | 46698580 | 7004787000 | 0.026 | 97.54 | 93.35 | 50.38 |
| ZH3 | 51892558 | 7783883700 | 0.0259 | 97.58 | 93.4 | 50.01 |
| ZHB1 | 51867412 | 7780111800 | 0.0261 | 97.46 | 93.2 | 51.1 |
| ZHB2 | 47901308 | 7185196200 | 0.0261 | 97.49 | 93.27 | 50.11 |

Abbreviations: OEB, OE rice with BPH feeding; OE, OE rice without BPH feeding; R, RNAi rice without BPH feeding; RB, RNAi rice with BPH feeding; ZH, WT without BPH feeding; ZHB, WT with BPH feeding.

**Table S2** Primer sequence of real-time quantitative PCR.

| Primer name | Sequence (5' to 3') |
| --- | --- |
| *OsPYL9-F* | CGCAACTACCGCTCCGTCAC |
| *OsPYL9-R* | TGGTGTCCTCCGCCGTGTTC |
| *OsAOS1-F* | CGGCGGCTTGCATCTCGTTC |
| *OsAOS1-R* | TTCGGCGACACCACCTCCTG |
| *OsJAZ11-F* | CGCTCTTCTACAACGGTTCAGTCG |
| *OsJAZ11-R* | GTTGCCTCGGTTGCCATCCTC |
| *OsbZIP23-F* | CTGTCGTCGCTGTCGCCATC |
| *OsbZIP23-R* | GATCATCCGCCGCTGTCTTCTC |
| *OsABA8ox2-F* | ACAGGGTGGAGGGCGAGATTG |
| *OsABA8ox2-R* | CTGCTTGGAGGCGAAGAAGACG |
| *Osbph6-F* | GTGCTGTCTCAAGTGGTGTCGTATAG |
| *Osbph6-R* | GACCGTAGAAATTCCATGCGTGTTTAG |
| *actin1-F* | CAGCACATTCCAGCAGAT |
| *actin1-R* | GGCTTAGCATTCTTGGGT |

**Table S3** Enriched genes involved in different pathway

| MSU ID | Gene name | Pathway | p-value | log_2_FC |
| --- | --- | --- | --- | --- |
| LOC_Os09g28390 | *OsABA8ox3* | Abscisic acid biosynthesis | 3.01*10^-4^ | 1.8754 |
| LOC_Os06g36670 | *OsPYL9* | Abscisic acid biosynthesis | 2.72*10^-5^ | 1.9763 |
| [LOC_Os03g08320](http://rice.uga.edu/cgi-bin/ORF_infopage.cgi?orf=LOC_Os03g08320) | *OsJAZ11* | Jasmonic acid biosynthesis | 6.48*10^-6^ | 2.3183 |
| LOC_Os03g55800 | *OsAOS1* | Jasmonic acid biosynthesis | 1.07*10^-24^ | 3.0281 |
| [LOC_Os04g35210](http://rice.uga.edu/cgi-bin/ORF_infopage.cgi?orf=LOC_Os04g35210) | *Osbph6* | Resistance to BPH | 1.499*10^-3^ | -1.271 |
| [LOC_Os02g52780](http://rice.uga.edu/cgi-bin/ORF_infopage.cgi?orf=LOC_Os02g52780) | *OsbZIP23* | ABA responsive element binding factor | 1.88*10^-4^ | 1.6172 |
